# In Vitro Study of Biocontrol Potential of Rhizospheric *Pseudomonas aeruginosa* against Pathogenic Fungi of Saffron (*Crocus sativus* L.)

**DOI:** 10.3390/pathogens10111423

**Published:** 2021-11-02

**Authors:** Shuang Hu, Xingxing Wang, Wenjing Sun, Lili Wang, Wankui Li

**Affiliations:** Institute of Chinese Materia Medica, Shanghai University of Traditional Chinese Medicine, Shanghai 201203, China; 15077877510@163.com (S.H.); wangxingrg@126.com (X.W.); sunwenjingyy@126.com (W.S.); biol2006@126.com (L.W.)

**Keywords:** *Pseudomonas aeruginosa*, saffron, rhizosphere, antagonistic activity, biological control, PGPR

## Abstract

Plant rhizosphere soil contains a large number of plant-growth promoting rhizobacteria, which can not only resist the invasion of pathogenic microorganisms and protect plants from damage, but also promote the growth and development of plants. In this study, *Pseudomonas aeruginosa* strain YY322, isolated and screened from the rhizosphere soil of saffron (*Crocus sativus* L.), was found through a plate confrontation experiment to show highly effectual and obvious antagonistic activity against the pathogens of saffron, including *Fusarium oxysporum*, *Fusarium solani*, *Penicillium citreosulfuratum*, *Penicillium citrinum* and *Stromatinia gladioli*. In addition, the volatile organic compounds of strain YY322 had great antagonistic activity against these pathogens. Observation under a scanning electron microscope and transmission electron microscope reflected that strain YY322 had a significant effect on the hyphae and conidia of *F. oxysporum* and *F. solani*. Through the detection of degrading enzymes, it was found that *P. aeruginosa* can secrete protease and glucanase. The plant growth promoting performance was evaluated, finding that strain YY322 had the functions of dissolving phosphorus, fixing nitrogen, producing siderophore and producing NH_3_. In addition, whole genome sequencing analysis indicated that the YY322 genome is comprised of a 6,382,345-bp circular chromosome, containing 5809 protein-coding genes and 151 RNA genes. The *P. aeruginosa* YY322 genome encodes genes related to phenazine (*phzABDEFGIMRS*), hydrogen cyanide(HCN) (*hcnABC*), surfactin (*srfAA*), salicylate (*pchA*), biofilm formation (*flgBCDEFGHIJKL*, *motAB*, *efp*, *hfq*), and colonization (*minCDE*, *yjbB*, *lysC*). These results collectively indicated the role of *P. aeruginosa* YY322 in plant growth enhancement and biocontrol mechanisms. All in all, this study provides a theoretical basis for *P. aeruginosa* as the PGPR of saffron, paving the way for the subsequent development and utilization of microbial fertilizer.

## 1. Introduction

Saffron (*Crocus sativus* L.) is a well-known dye and spice, widely cultivated in India, Iran, China, Egypt, France, Greece, Israel, Italy, Spain, Turkey and other countries [1]. Saffron is an essential medicinal plant in China, purported to have the ability of promoting blood circulation, removing blood stasis, cooling blood, resolving toxins, relieving depression, and calming nerves. However, because of its low output and high values, saffron is costly and is referred to as “red gold” [2,3]. At present, saffron is widely cultivated on Chongming Island in Shanghai. However, many corms have rotted due to long-term continuous cropping and other factors, resulting in a decline in saffron yield and quality. Although using chemical fertilizers and pesticides reduces the occurrence and development of diseases, they inevitably contribute to environmental pollution and safety issues with medicinal materials.

The term rhizosphere, coined by German scientist Lorenz Hiltner in 1904, refers to a micro-domain environment influenced by plant root activities and differs from soil in physical, chemical, and biological properties. It is considered as the second genome of plants and is a hotspot for plant–soil–microbe interactions [4,5,6]. Due to unique environment of rhizosphere soil, numerous microorganisms gather in this area, where they play a vital role in the life history of plants [7,8]. Numerous plant growth-promoting rhizobacteria (PGPR) capable of strengthening plant nutrient absorption and immunological function have been isolated from rhizosphere soil, and a number of these have been converted into microbial fertilizers with favorable results [9,10,11].

Currently, the most extensively investigated PGPR includes *Pseudomonas*, *Bacillus*, *Agrobacterium*, *Eriwinia*, *Flavobacterium*, *Pasteuria*, *Serratia*, *Enterobacter*, etc. *Pseudomonas* is a type of obligate aerobic gram-negative rod-shaped bacteria widely distributed in nature and has garnered widespread interest due to its intimate association with human activities. *Pseudomonas* grows rapidly and can adapt to the natural environment. Aside from this, it can effectively antagonize the occurrence and development of plant pathogens by secreting a series of metabolites and can help plants to absorb and utilize nutrients [12]. In addition, it is easily selected and bred through genetic mutation or modification, then inoculated into plants’ rhizospheres using bacterialization of seeds to perform its function [13]. Currently, the main types of *Pseudomonas* classified as PGPR are *P. putida* [14], *P. aeruginosa* [15], *P. fluorescens* [16,17], *P. chlororaphis* [18], and *P. Parafulva* [19].

Herein, 85 strains of bacteria were isolated from the rhizosphere soil of saffron. Among them, strain YY322 exhibited a good antagonistic effect against the five pathogens of saffron. Combining physiological and biochemical tests and molecular biology identification, it was identified as *P. aeruginosa*. The strain YY322 could destroy the hyphae and conidia of pathogens, as observed under a scanning electron microscope (SEM) and transmission electron microscope (TEM). Moreover, hydrolytic enzymatic (chitinase, protease, cellulase, and glucanase) activity and plant growth-promoting (PGP) potential (phosphate solubilization, potassium dissolution, atmospheric nitrogen fixation, siderophore production, indole-3-acetic acid (IAA) and ammonia (NH_3_) production, 1-aminocyclopropane-1-carboxylate (ACC) deaminase activity) of the strain were tested, discovering that the strain possessed good performance. Finally, we performed whole-genome sequencing of the bacterium to further explore its antagonistic and growth-promoting mechanisms. This article provides evidence to prove that *P. aeruginosa* can be used as PGPR in the production of saffron, laying a theoretical foundation for the development and utilization of microbial fertilizer.

## 2. Results

### 2.1. Isolation and Screening of Antagonistic Bacteria

**Figure 1:**
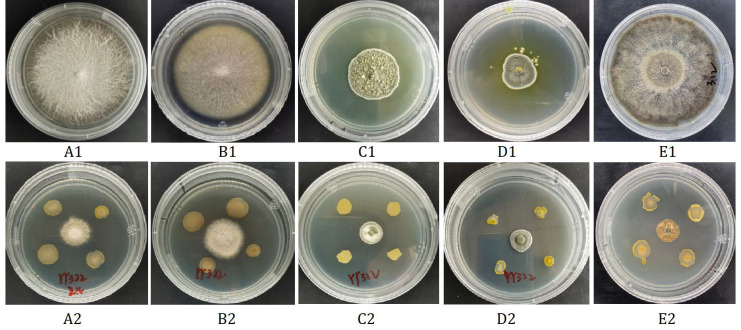
Antagonistic effect of strain YY322 on five pathogens cultivated on PDA medium for one to two weeks. (**A**) *F. oxysporum,* (**B**) *F. solani,* (**C**) *P. citreosulfuratum,* (**D**) *P. citrinum,* (**E**) *S. gladioli* (**1**) control, (**2**) treatment.

A total of 85 strains of bacteria were isolated from saffron rhizosphere soil. After several screenings, it was found that the antagonistic effect of strain YY322 was the best, with inhibitory rates for *Fusarium oxysporum*, *Fusarium solani*, *Penicillium citreosulfuratum*, *Penicillium citrinum*, and *Stromatinia gladioli* of 73.17%, 62.20%, 23.16%, 49.17%, and 79.76%, respectively (Figure 1). During the process of plate confrontation, not only was the growth of pathogenic fungi significantly inhibited, but the morphological characteristics of strain YY322 were also changed compared with the normal state, indicating that it may have produced some unknown antifungal substances. The strain is currently stored in the China Center for Type Culture Collection under the number CCTCC M 2021208 YY322.

### 2.2. Strain YY322: Fusarium *spp.* Interaction Study by SEM and TEM

**Figure 2:**
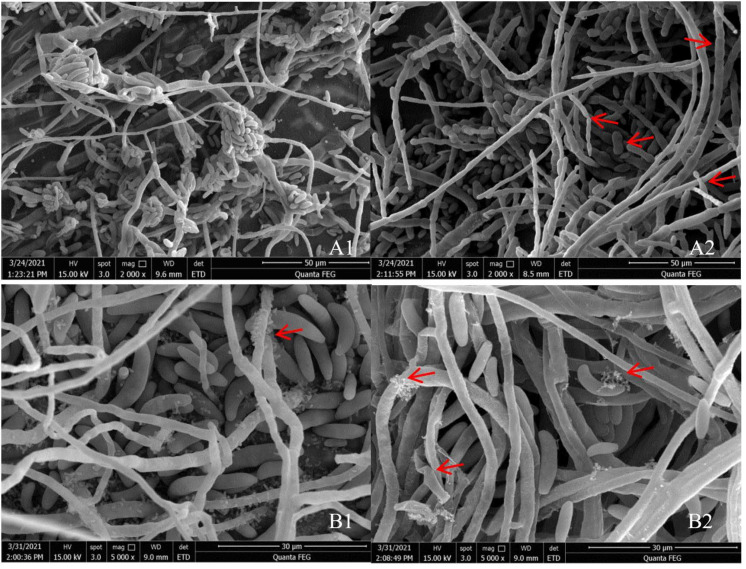
Observation of the morphological changes of hyphae and conidia under SEM. (**A**) *F. oxysporum;* (**B**) *F. solani* (**1**) control: the hyphae and conidia showed normal growth, (**2**) treatment: the surface of the hyphae is shrunk and broken, and its contents leak out; the conidia develop abnormally and decrease in number (arrowheads).

**Figure 3:**
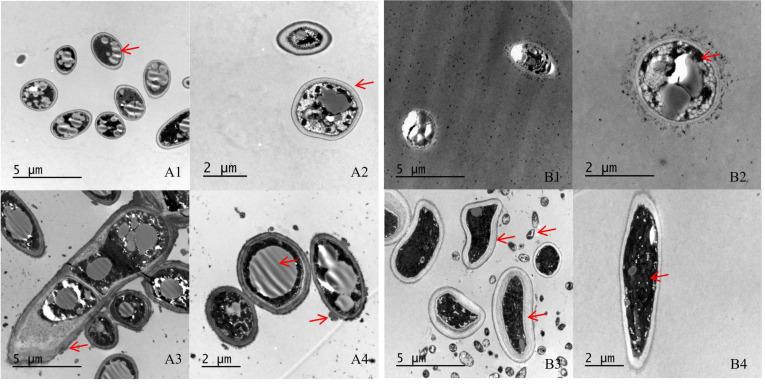
Observation of the changes in the internal structure of hyphae under TEM. (**A**) *F. oxysporum;* (**B**) *F. solani* (**1**,**2**) control: the evenly distributed hyphae have clear boundaries, the cell wall and cell membrane structure are complete, and the cytoplasm is in a normal state, (**3**,**4**) treatment: the section of hyphae is irregular, the cell wall is thickened, the cytoplasm is turbid and chaotic, and the vacuole develops abnormally (arrowheads).

SEM indicated that the hyphae and conidia of *Fusarium* spp. under the action of strain YY322 were changed significantly compared with the control group. Hyphae were round and full in the control group, with complete morphology and structure, smooth surface, and uniform thickness, whereas conidia were normal spindle-shaped, moderate in size, and demonstrating a good growth state. *F. oxysporum* affected by strain YY322 had numerous broken hyphae, with an uneven surface, severe segmentation, abnormal apex development, and malformed conidia. *F. solani* affected by strain YY322 caused seriously damaged hyphae, with surface shrinkage and leakage of contents. In addition, it possessed only a few large conidia and no small conidia (Figure 2). Under TEM, it could be observed that compared with the control group, the internal structure of Fusarium spp. under the action of strain YY322 was severely damaged. In the control group, the evenly distributed hyphae were regularly round and exhibited clear boundaries. The cell wall and membrane remained intact and compact, and the cytoplasm was well-distributed, maintaining a normal growth state. The cell walls of the hyphae of *F. oxysporum* affected by strain YY322 ruptured, resulting in leakage of the contents. The number of vacuoles decreased, but their volume increased. In addition, the cytoplasm was turbid and chaotic. *F. solani*, affected by strain YY322, exhibited numerous immature conidia gathered together, and the shape of the hyphae section was abnormal. The cell wall was thickened, the cytoplasm was mixed and black, and the vacuole almost disappeared (Figure 3).

### 2.3. Inhibitory Effect of VOCs of Strain YY322 on Mycelial Growth

**Figure 4:**
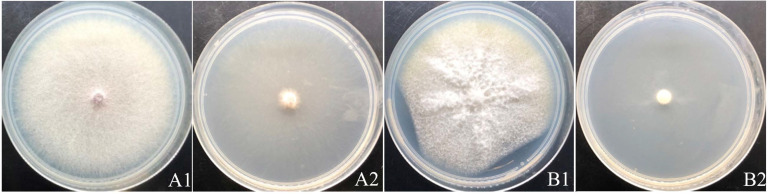
Antifungal activity of VOCs: (**A**) *F. oxysporum;* (**B**) *F. solani* (**1**) control, (**2**) treatment.

Since we discovered that the strain YY322 could produce a special smell when cultured, we tested the antifungal effect of its VOCs, and the results showed it demonstrated a pronounced inhibitory effect on *F. oxysporum* and *F. solani*, with inhibition rates of 90.24% and 89.02%, respectively. Intriguingly, a few hyphae of both pathogens could still be observed spreading to the entire plate (Figure 4).

### 2.4. Identification of Strain YY322

**Figure 5:**
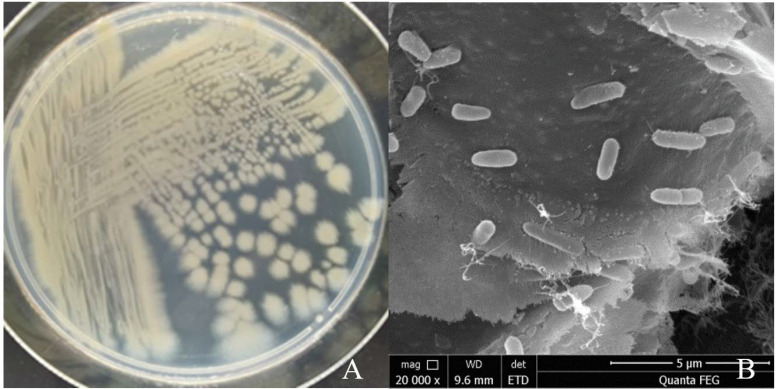
Morphology of strain YY322: (**A**) colony morphology and (**B**) microscopic morphology.

**Figure 6:**
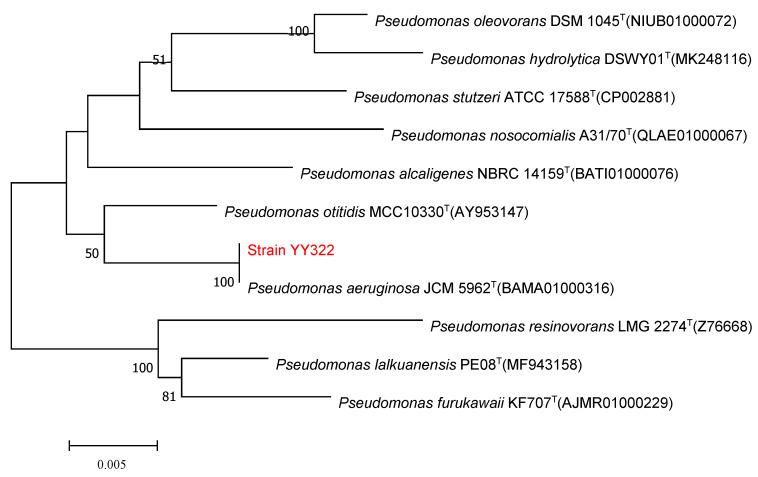
16S rDNA sequence phylogenetic tree of strain YY322: the numbers at the nodes indicate the levels of bootstrap support (%) based on 1000 reassembled datasets, in which only the branches with >50% bootstrap support are labeled, and the superscript “T” indicates the model strain.

The single colony of strain YY322 on LB medium after being incubated for 24 h at 30 °C was nearly round, relatively flat, light yellow-green, not smooth, and opaque with irregular edges (Figure 5A). Under SEM, the strain YY322 was observed to be rod-shaped, with a size of about 0.5 µm × 2 µm (Figure 5B). Subsequently, the strain YY322 was identified as *P. aeruginosa* according to its 16S rDNA sequence phylogenetic tree (Figure 6), deposited in GenBank under accession number MZ026485. Finally, according to physiological and biochemical tests, strain YY322 was confirmed as *P. aeruginosa* (Table 1).

### 2.5. Hydrolytic Enzymes Activities of Strain YY322

According to the experimental phenomenon, strain YY322 exhibited strong protease activity and weak glucanase activity but lacked cellulase and chitinase activities (Supplementary materials, Figure S1).

### 2.6. Screening of Plant Growth-Promoting Attributes of Strain YY322

The strain YY322 could fix nitrogen, solubilize organic and inorganic phosphate, and produce siderophore and NH_3_, but did not dissolve potassium or produce IAA and ACC deaminase enzyme (Supplementary Materials, Figure S2).

### 2.7. WGS Analysis of Strain YY322

**Figure 7:**
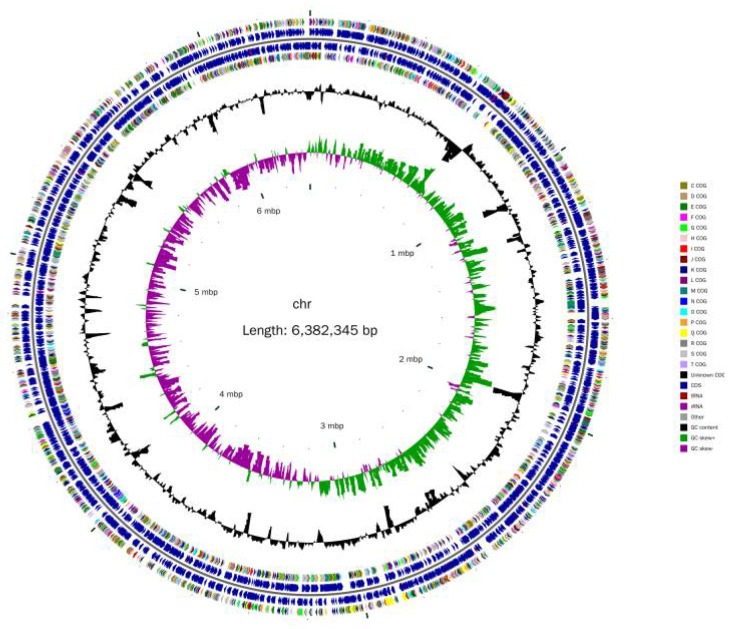
YY322 genome circle: from inside to out, the first circle represents the scale, the second circle represents GC Skew, the third circle represents GC content, the fourth and seventh circles represent the COG (Cluster of Orthologous Groups of proteins) to which each CDS (Coding sequence) belongs, and the fifth and sixth circles represent the position of CDS, tRNA, and rRNA in the genome.

The complete genome of YY322 was composed of a 6,382,345 bp circular chromosome with a 66.47% GC content (GenBank number: CP080518, Figure 7), which contained 5809 protein-coding genes (CDSs), 64 tRNA genes, 12 rRNA genes, and 75 other ncRNA. CRISPR analysis forecast seven sequences with confirmed type. Detailed information is provided in the Supplemental Data Supplementary materials (Tables S1–S3).

**Figure 8:**
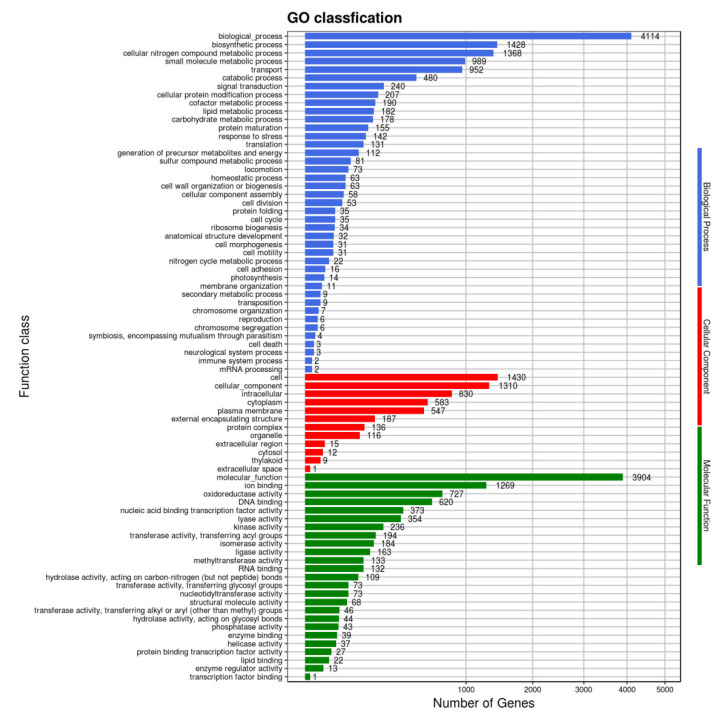
Clusters of GO annotation.

**Figure 9:**
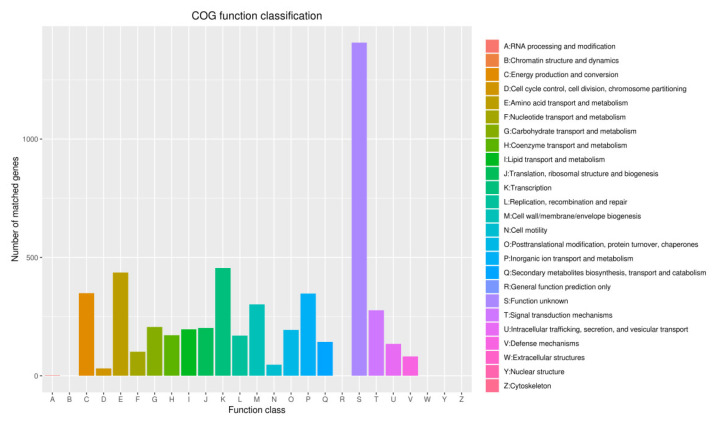
Clusters of COG annotation: Number of matched genes—(A) 2, (B) 0, (C) 349, (D) 31, (E) 436, (F) 102, (G) 206, (H) 171, (I) 196, (J) 202, (K) 455, (L) 170, (M) 302, (N) 45, (O) 194, (P) 347, (Q) 143, (R) 0, (S) 1407, (T) 277, (U) 135, (V) 82, (W) 0, (Y) 0, (Z) 0.

**Figure 10:**
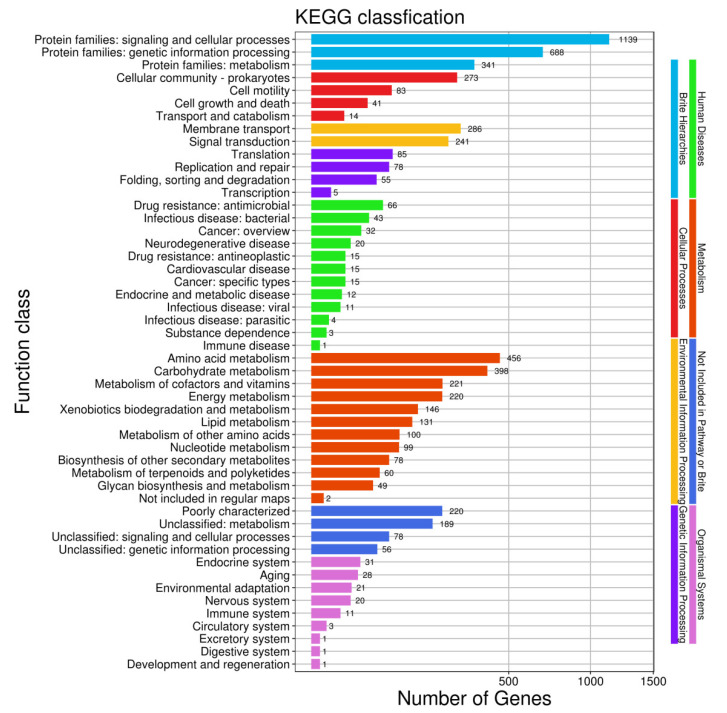
Clusters of KEGG annotation.

In the comparison of different databases, there were 4377 protein-encoding genes in the GO database, 5254 protein-encoding genes in the eggNOG database, and 3192 protein-encoding genes in the KEGG database. GO analysis was used to categorize genes into three categories according to matches with known sequences. The seven main pathways were biological process (GO: 0008150, 4114 genes), molecular function (GO: 0003674, 3904 genes), cell (GO: 0005623, 1430 genes), biosynthetic process (GO: 0009058, 1428 genes), cellular nitrogen compound metabolic process (GO: 0034641, 1368 genes), cellular component (GO: 0005575, 1310 genes) and ion binding (GO: 0043167, 1269 genes) (Figure 8). The eggNOG classification annotation showed that 90.45% of protein-encoding genes could be annotated to eggnog; however, the number of unknown function genes was the highest and up to 1407, accounting for 24.22% of total protein-encoding genes. Among these known functional genes, the genes for transcription were the most abundant, with 455 genes accounting for 7.83%, followed by 436 genes for regulating amino acid transport and metabolism, accounting for 7.51% (Figure 9). Of the eight classifications of KEGG pathways, brite hierarchies (protein families: signaling and cellular processes, genetic information processing, metabolism) contained the highest number of genes, followed by metabolism (amino acid and carbohydrate metabolism) (Figure 10).

**Figure 11:**
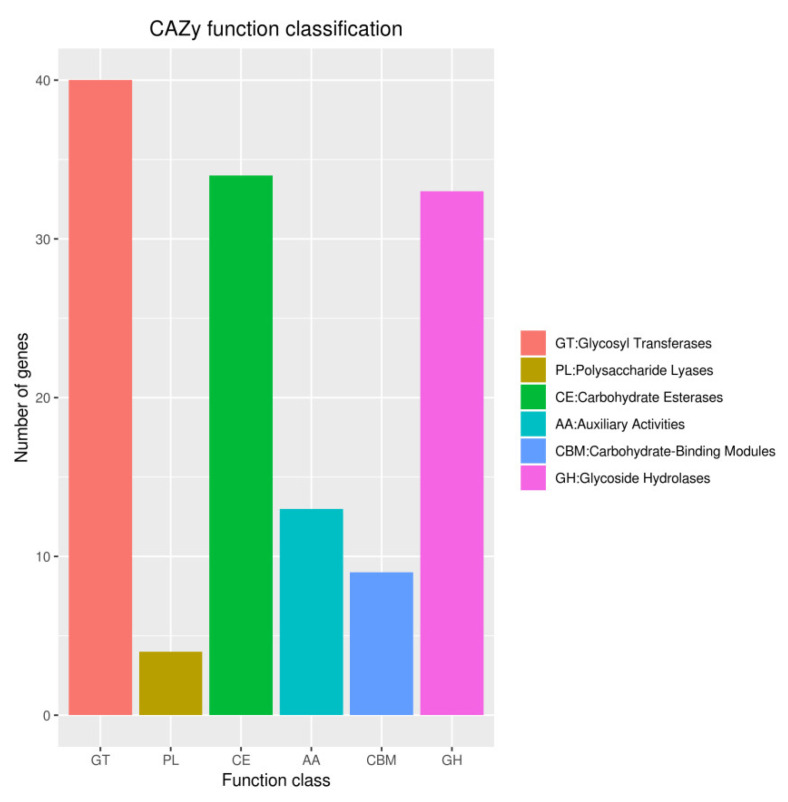
Gene count distributions of carbohydrate-active enzyme families.

The CAZymes analysis revealed that the glycosyl transferase (GTs; 40 genes) family was the major category, followed by carbohydrate esterases (CEs; 34 genes), and glycoside hydrolases (GHs; 33 genes) (Figure 11). Data obtained from antiSMASH 6.0.1 showed strain YY322 encodes 15 secondary metabolite biosynthesis gene clusters (BGCs), including four NRPS (non-ribosomal peptide synthetase), two NRPS-like, two RiPP (other unspecified ribosomally synthesized and post-translationally modified peptide product cluster), two phenazine, two hserlactone, one NAGGN (N-acetylglutaminylglutamine amide), one T1PKS (type I polyketide synthase), one T3PKS (type III polyketide synthase), one redox-cofactor, one thiopeptide, and one betalactone. Three of these shared 100% similarity with the accession sequence, which encoded coelibactin, pyocyanine and L-2-amino-4-methoxy-trans-3-butenoic acid (Table 2).

## 3. Discussion

As a traditional Chinese medicine in China, saffron has been revealed by many researchers to have remarkable benefits as a result of current research advancements [20,21]. To meet the domestic market demand, the cultivation area of saffron has gradually expanded. However, due to factors such as corm origin, planting technology, and climate environment, saffron diseases have become increasingly prevalent, resulting in massive losses to farmers and impeding the saffron industry’s continued development [22]. At present, the most pathogenic microorganism for saffron is *F. oxysporum*. Once it is allowed to multiply and expand in the field, it has a devastating impact on saffron growth. *F. oxysporum*, one of the top ten plant pathogenic fungi, is a ubiquitous soil-borne pathogen that leads to vascular wilt on a wide range of plants [23]. Although chemical pesticides have significantly reduced the infection of pathogens such as *F. oxysporum*, they leave pesticide residues and may affect the quality of saffron medicinal materials. Thereby, it is imperative to seek new technical means to inhibit these pathogenic fungi in order to achieve the purpose of protecting saffron.

The discovery of PGPR with antagonistic and PGR effects from the rhizosphere has become a current research focus. Plants can release chemical signals in response to their own requirements, which significantly influence the composition and function of rhizosphere soil microorganisms, contributing to promoting plant growth and resisting biotic and abiotic stress. Yuan’s study suggested that, when exposed to aboveground pathogens, plants will recruit beneficial rhizosphere communities by modification of plant exudation patterns so as to benefit subsequent plant generations [24]. As a result, the rhizosphere of healthy plants usually contains a lot of PGPR. Yang’s research indicated that seven PGPR from the field-grown barley rhizosphere could increase plant growth promotion and biocontrol of *Fusarium* wilt in watermelon [11].

In this study, strain YY322 was isolated from the rhizosphere soil of healthy saffron and had a noticeable antagonistic effect on the five pathogenic fungi of saffron. According to its morphological and biochemical characteristics and 16S rRNA gene sequencing, strain YY322 was identified as *P. aeruginosa*. *P. aeruginosa* is widely distributed in the natural environment; it is a conditional pathogen for humans, particularly for burn patients, cancer patients, immunodeficiency patients, and cystic fibrosis patients. Recent research has demonstrated that it can also be utilized as PGPR. Chandra discovered that *P. aeruginosa* isolated from the rhizosphere of *Valeriana wallichi* was subjected to in vitro biocontrol activity against *F. oxysporum*, *Alternaria alternata,* and *Aspergillus flavus* and displayed plant growth promotion rhizobacterial activity [15]. Singh discovered that as an effective colonizer in sugarcane, *P. aeruginosa* strain B18 could regulate plant hormone production and enhance host-plant resistance to smut pathogen *S. scitamineum* in a smut-susceptible sugarcane variety to improve growth [25]. Sun proved that the salt-tolerant PCN-producing *P. aeruginosa* NF011 had the possibility of controlling wheat fungal disease [26].

Previous studies have demonstrated that the main mechanisms of antagonistic antibacterial action include direct secretion of antibacterial substances, competition between nutrition and space, and induction of plant system resistance. This study revealed that strain YY322 produces protease and glucanase, consequently destroying the cell wall of pathogenic fungi, as observed under SEM and TEM, similar to the experimental result of Al-Ghafri [27]. In addition, combined with the antibacterial testing of VOCs, strain YY322 markedly inhibits hyphae development and conidia production, thus effectively controlling growth and reproduction of pathogens. Moreover, WGS analysis showed that strain YY322 contains *phzABDEFGIMRS* genes, which prove it can produce phenazines with obvious antifungal effects. Liu’s study demonstrated that 1-hydroxyphenazine from *P. aeruginosa* could strongly inhibit the growth of plant pathogenic fungi and bacteria [28]. In addition, strain YY322 could endow host plants with resistance to pathogens, as HCN (*hcnABC*), surfactin (*srfAA*), and salicylate (*pchA*) genes were found in its genome [29,30].

PGP performance evaluation tests found that strain YY322 could fix nitrogen, dissolve phosphorus, and produce siderophore and NH_3_. The nitrogen on the earth’s surface mainly exists as N_2_, but plants cannot directly utilize nitrogen in N_2_. As a result, nitrogen deficiency is a prevalent problem in farmland soils in China, and it is one of the main factors restricting agricultural production efficiency [31]. The ability to fix nitrogen and produce NH_3_ of strain YY322 can solve this problem to a certain extent. Similarly, we found its genome contains nitrogen metabolism-related genes, including *glnGL*, *aatJMPQ*, *gltBDPST,* and *norBCDERQ*, proving its nitrogen-fixing ability. Phosphorus is a critical nutrient indispensable for plant life activity, but the effective utilization rate of phosphate fertilizer is low, and its production requires much energy, causing environmental pollution [32]. Phosphorus deficiency in farmland soil is a widespread concern faced by most countries worldwide. Strain YY322 can dissolve insoluble phosphorus in the soil through its own metabolism, converting it into effective phosphorus that plants can absorb and utilize. In our study, strain YY322 genomic sequence analysis also confirmed the existence of *pstABCS*, *phoABDHRU,* and *phnCDEFGHIJKLMNP* genes, which are responsible for phosphate metabolism. Iron is ubiquitous and abundant in soil, but most iron in soil is insoluble iron oxide, which is challenging to use by plants, so plants frequently suffer from iron deficiency [33]. In view of the result of plate experiments and the discovery of *fes*, *fepA*, *mbtH*, *cirA*, *fpvA*, *entD*, *acrAB*, and *serABCS* genes, it is shown that strain YY322 can produce and release siderophores, which chelate ferric ions in soil and form Fe^3+^-siderophore complexes. They can enter the plant body through a particular plant and microbial cell membrane channel and then be reduced to divalent iron (Fe^2+^), increasing the plant’s use of insoluble iron in soil. Concurrently, it also can resist the competition of pathogens for iron and inhibit the reproduction of pathogens [34]. Furthermore, we found the genes (*efp*, *hfq*, *motAB*, *flgBCDEFGHIJKL*) involved in biofilm formation and the genes (*minCDE*, *lysC*, *yjbB*) involved in colonization in the genome of strain YY322. The formation of biofilm is conducive to the adaptation of microorganisms to poor environments and is closely related to the colonization ability of biocontrol bacteria, which can effectively resist the invasion of pathogens [35].

## 4. Materials and Methods

### 4.1. Pathogenic Strains for Antagonistic Test

Five saffron pathogens (*F*. *oxysporum* [36], *F*. *solani* [36], *P*. *citreosulfuratum* [37], *P*. *citrinum* [36], and *S*. *gladioli* [38]), isolated and identified from diseased saffron corms by our research team in 2020, were stored in a refrigerator at 4 °C.

### 4.2. Sampling Process and Bacterial Isolation

In December 2020, rhizosphere soil samples were collected from Chongming district (31.62° N, 121.40° E), Shanghai, China, for isolating rhizobacterial strains. The corms were uprooted, and the soil adhering to the roots was collected in sterile plastic bags, resembling rhizosphere soil [39]. The soil samples were then cold transferred to the Institute of Chinese Materia Medica at Shanghai University of Traditional Chinese Medicine for immediate processing. The rhizosphere soil was crushed and sieved after being dried in the shade for two weeks. Around 5 g of rhizosphere soil was mixed with 45 mL of sterile distilled water in a flask and shaken on a rotary shaker at 30 °C for 2 h. Following that, 1 mL of flask suspension was added to a 10 mL vial and diluted to 10^−^^3^, 10^−^^4^, and 10^−^^5^. Approximately 0.2 mL of each suspension was spread separately on Luria-Bertani (LB) media plates altered with 50 μg/mL cycloheximide to inhibit fungi growth. After that, the plates were placed upside down at 30 °C for 48 h, and well-isolated single colonies with different morphological characteristics were selected for streaking on fresh LB media plates to obtain the pure culture, which was then stored on LB media slants at 4 °C in a refrigerator for subsequent experiments.

### 4.3. Screening of Antagonistic Bacteria

The isolated strains were screened in vitro on potato dextrose agar (PDA) medium using the dual-culture method [40]. *F. oxysporum*, *F. solani,* and *S. gladioli* were inoculated on PDA medium and activated at 28 °C for seven days. *P. citrinum* and *P. citreosulfuratum* were inoculated on PDA medium through the streaking method of partition and activated at 28 °C for two days to acquire single colonies. Meanwhile, the antagonistic bacteria obtained from preliminary screening were inoculated on LB medium and cultured at 30 °C for 24 h. Then, using a puncher, 5 mm diameter pathogen plugs were obtained and placed in the center of PDA plate, while bacteria were spotted 25 mm around the plug using sterile toothpicks. PDA plates only with the pathogen plug were employed as controls. All plates were kept at 28 °C for one to two weeks. Each treatment was replicated three times, and the experiment was repeated twice to determine the best antagonistic bacterium by calculating the inhibition rate. Percentage inhibition was calculated as follows: Inhibition rate = ((control colony diameter − treated colony diameter)/control colony diameter) × 100% [41].

### 4.4. Electron Microscopic Analysis

#### 4.4.1. Observation of the Effect of the Best Antagonistic Bacterium on *F. oxysporum* and *F. solani* by SEM

*F. oxysporum* and *F. solani* were cultured on PDA medium for ten days according to the above-mentioned method with or without bacterium, after which *F. oxysporum* and *F. solani* samples were collected for SEM. The fungal samples were fixed in 2.5% (*v*/*v*) glutaraldehyde for 24 h, rinsed with 0.1 M phosphate buffer (pH 7.2) three times (every time for 15 min), fixed in 1% (*w*/*v*) osmic acid for 3 h, rinsed with 0.1 M phosphate buffer (pH 7.2) three times (every time for 15 min), and dehydrated in an ascending alcohol concentration series (30%, 50%, 70%, 90%, 100% three times, for 15 min each). The samples were dried using a Leica EM CPD300 critical point dryer (Germany) and coated with gold using a Leica EM ACE600 ion sputter coater (Austria). A QUANTA FEG250 scanning electron microscope (Netherlands) was deployed for observing sputter-coated samples.

#### 4.4.2. Observation of the Effect of the Best Antagonistic Bacterium on *F. oxysporum* and *F. solani* by TEM

The fungal samples were cut into 1 mm^3^ pieces by a disposable blade, fixed with 2.5% (*v*/*v*) glutaraldehyde, and stored at 4 °C. Next, they were rinsed with 0.1 M phosphate buffer (pH 7.2) three times (every time for 15 min), fixed in 1% (*w*/*v*) osmic acid for 2 h, rinsed with 0.1 M phosphate buffer (pH 7.2) three times (every time for 15 min), and dehydrated in 30% ethanol for 15 min, 50% ethanol for 15 min, 70% ethanol-uranyl acetate overnight, 90% ethanol for 15 min, 90% ethanol:90% acetone (1:1) for 15 min, 90% acetone for 15 min, and 100% acetone 3 times (every time for 15 min). After that, they were polymerized with acetone:resin (2:1) for 4 h, acetone:resin (1:2) overnight, pure resin twice (every time for 2 h), and transferred to the embedding frame for fixation at 37 °C for 24 h and 60 °C for 48 h. Finally, they were sliced into 70 nm slices using Leica 705902 ultra-thin microtome (Germany), stained with lead citrate for 15 min, rinsed three times with dd H_2_O, and dried naturally before being examined using FEI Tecnai G2 Spirit TEM.

### 4.5. The Effect of Volatile Organic Compounds (VOCs) of the Best Antagonistic Bacterium on F. oxysporum and F. solani

In order to assess the production of antifungal VOCs by the bacterium, the double plate assay method [42] was employed, with slight modifications. An appropriate amount of LB medium was poured on the plate’s cover and coated with bacterium that was cultivated for 24 h. Concurrently, an appropriate amount of PDA medium was poured on another part of the plate, and a 5 mm pathogen plug was placed in the center. After that, two parts of the plate were sealed together with parafilm and plastic wrap, and all plates were placed upside down at 28 °C for seven days. The control treatment was a PDA plate inoculated with only a 5 mm pathogen plug. Each treatment was replicated three times. The percentage of pathogen growth inhibition caused by VOCs produced by bacterium was determined from the above formula.

### 4.6. Identification of the Best Antagonistic Bacterium

#### 4.6.1. Morphological, Physiological, and Biochemical Characterizations

The morphology of bacterium was observed using SEM with the same method as above. The physiological and biochemical characterizations (gram reaction, growth at 4 °C and 41 °C, oxidase, catalase, urease, lipase, MR, V-P, gelatin liquefaction, starch hydrolysis, milk coagulation, H_2_S production, nitrate reduction, and citrate utilization) of bacterium were tested on the basis of “Bergey’s Manual of Determinative Bacteriology” [43].

#### 4.6.2. Molecular Identification

The DNA of bacterium was extracted using FastPure Bacteria DNA Isolation Mini kit (Vazyme Biotech Co., Ltd., Nanjing, China) according to the manufacturer’s instructions. The 16S rRNA gene sequence was amplified using the primers 27F: 5′-AGAGTTTGATCCTGGCTCAG-3′ and 1492R: 5′-TACCTTGTTACGACTT-3′ [44]. PCR amplifications were conducted in ABI-2720 PCR thermal cycler (Applied Biosystems, CA, USA) as follows: one cycle of 5 min at 95 °C; followed by 35 cycles of 30 s at 95 °C, 30 s at 55 °C, and 1 min at 72 °C; then followed by one cycle of 5 min at 72 °C. The amplified PCR product was then purified and Sanger sequenced at Personal Biotech Co., Ltd. (Shanghai, China). The obtained 16S rDNA sequence of bacterium was examined by the NCBI database using BLAST algorithms at National Center for Biotechnology Information website (https://www.ncbi.nlm.nih.gov/BLAST, accessed on 26 April 2021) [45]. The phylogenetic tree was constructed using the Neighbor-Joining method with MEGA 7.0 software.

### 4.7. Detection of Hydrolytic Enzyme Activities

Since enzymatic cell wall degradation is a potential mechanism for inhibiting fungal growth, the agar diffusion method was used to evaluate whether the bacterium has extracellular hydrolytic enzymes [46]. We utilized chitinase indicator medium (per liter: MgSO_4_·7H_2_O, 0.2 g; KCl, 0.2 g; NH_4_H_2_PO_4_, 1 g; colloidal chitin, 10 g; agar, 18 g), protease indicator medium (per liter: skimmed milk, 15 g; agar, 18 g), cellulase indicator medium (per liter: Congo red, 0.4 g; MgSO_4_·7H_2_O, 0.5 g; NaCl, 0.5 g; KH_2_PO_4_, 1 g; (NH_4_)_2_SO_4_, 2 g; CMC-Na, 2 g; agar, 18 g) and glucanase indicator medium (per liter: FeSO_4_, 0.01 g; Congo red, 0.05 g; KCl, 0.5 g; MgSO_4_, 0.5 g; K_2_HPO_4_, 1 g; NaNO_3_, 2 g; β-glucan, 2 g; agar, 18 g). The bacterium was spotted on the above-mentioned medium with a sterile toothpick and incubated at 30 °C for 48 h. A distinct halo was observed surrounding colonies grown on specific agar media, testifying to the existence of extracellular hydrolytic enzymes. Each experiment was replicated three times.

### 4.8. Assessment of PGP Potential

Phosphate solubilization was qualitatively determined using Esmaeel’s methodology [47]. Potassium dissolution was qualitatively determined using Wang’s methodology [48]. Atmospheric nitrogen fixation was qualitatively determined using Estrada-De Los Santos’s methodology [49]. ACC deaminase enzyme production was qualitatively determined using Penrose and Glick’s methodology [50]. IAA production was qualitatively determined using Dowarah’s methodology [51]. NH_3_ production was qualitatively determined using Dutta and Thakur’s methodology [52]. Siderophore production was qualitatively determined using Dukare’s methodology [53]. Each experiment was replicated three times.

### 4.9. Whole Genome Sequencing (WGS) of the Best Antagonistic Bacterium

The bacterium was inoculated into a clear and transparent LB liquid medium and cultured in a constant temperature shaker at 200 rpm at 30 °C. When the OD600 was about 0.6, the bacterial solution was centrifuged at 8000 rpm for 5 min at 4 °C to collect the bacterial cells. Then the cells were gently washed with an appropriate amount of PBS buffer, and the supernatant was removed completely. The cells were quick-frozen with liquid nitrogen for 15 min and then cold-transported to Personal Biotech Co., Ltd. (Shanghai, China) for whole-genome sequencing analysis.

The genomic DNA was extracted using the Cetyltrimethyl Ammonium Bromide (CTAB) method with minor modifications, after which the DNA concentration, quality, and integrity were determined by a Qubit Flurometer (Invitrogen, Carlsbad, CA, USA) and a NanoDrop Spectrophotometer (Thermo Fisher Scientific, Waltham, MA, USA). Sequencing libraries were generated by the TruSeq DNA Sample Preparation Kit (Illumina, Ipswich, CA, USA) and the Template Prep Kit (Pacific Biosciences, Menlo Park, CA, USA). The genome sequencing was then performed by the Pacific Biosciences platform and the Illumina Miseq platform. Data assembly proceeded after adapter contamination removal and data filtering using AdapterRemoval [54] and SOAPec [55]. The filtered reads were assembled by SPAdes [56] and A5-miseq [57] to construct scaffolds and contigs. Canu [58] software was applied to assemble the data acquired by Pacbio platform sequencing. Afterwards, whole assembled results were integrated to generate a complete sequence, and the genome sequence was obtained after correction using pilon software [59].

Genome function element prediction contained the forecast of coding-gene, non-coding RNA, and clustered regularly interspaced short palindromic repeats (CRISPRs). Gene prediction was performed by Glimmer 3.02 [60]. tRNAscan-SE [61], RNAmmer [62], and Rfam [63] were used to discover tRNA, rRNA, and other ncRNA, respectively. CRISPRs were identified by the CRISPR recognition tool [64]. Function annotation was completed by blast search against GO (Gene Ontology) [65], COG (Cluster of Orthologous Groups of proteins) [66], and KEGG (Kyoto Encyclopedia of Gene and Genomes) [67]. Subsequently, Carbohydrate-Active enzymes and Bioactive secondary metabolites were predicted with CAZymes (Carbohydrate-Active enzymes) database [68] and antiSMASH 6.0.1 (https://antismash.secondarymetabolites.org/#!/start, accessed on 10 October 2021), respectively. Finally, CGview [69] was used to give an overview of the genome information.

## 5. Conclusions

In summary, in the light of this study, we consider that *P. aeuroginosa* could be employed as a biocontrol agent against pathogenic fungi of saffron, and preliminary tests indicate that it possesses PGPR traits, consistent with Islam’s conclusion [70]. It is worth mentioning that this is the first report that *P. aeruginosa* has an antagonistic effect on *P. citreosulfuratum*, *P. citrinum*, and *S. gladioli*. In consideration of the above results, we are convinced that strain YY322 can be developed into a saffron microbial fertilizer. We plan to further verify the antagonistic and PGP effects of strain YY322 in indoor and field trials. Meanwhile, more extensive and in-depth research is required to overcome future problems and challenges with regard to saffron production.

## Figures and Tables

**Table 1:** Morphological, biochemical, plant growth-promoting, and antagonistic activity of strain YY322.

Physiological and Biochemical Characterization	Result	Hydrolytic Enzyme Activities and PGP Attributes	Result
gram reaction	−	chitinase	−
growth at 4 °C	−	protease	+
growth at 41 °C	+	cellulase	−
oxidase	+	glucanase	+
catalase	+		
urease	+	phosphate solubilization	+
lipase	+	potassium dissolution	−
MR	−	nitrogen fixation	+
V-P	−	ACC deaminase enzyme	−
gelatin liquefaction	+	IAA production	−
starch hydrolysis	−	NH_3_ production	+
milk coagulation	+	siderophore production	+
H_2_S production	−		
nitrate reduction	+		
citrate utilization	+		

Abbreviations: +, positive for test; −, negative for test.

**Table 2:** Results of the search for antimicrobial gene clusters in the genome of strain YY322.

Region	Type	From	To	Most Similar Known Cluster	Similarity
1	NRPS, phenazine	738,712	795,458	marinophenazine A/phenaziterpene A	30%
2	RiPP-like	909,671	920,361	–	–
3	NRPS-like	926,710	969,065	–	–
4	hserlactone	1,612,259	1,632,202	–	–
5	NAGGN	1,635,525	1,650,285	–	–
6	NRPS	1,753,238	1,800,066	–	–
7	RiPP-like	1,828,970	1,839,824	–	–
8	T3PKS,T1PKS	2,602,677	2,661,176	pyoluteorin	100%
9	NRPS	2,835,654	2,949,679	pyoverdin	24%
10	NRPS	3,021,594	3,073,805	L-2-amino-4-methoxy-trans-3-butenoic acid	100%
11	redox-cofactor	3,422,524	3,444,653	lankacidin C	13%
12	thiopeptide	3,493,168	3,526,171	oxalomycin B	6%
13	phenazine	3,528,349	3,549,361	pyocyanine	100%
14	hserlactone	4,040,057	4,060,662	–	–
15	NRPS-like, betalactone	4,255,904	4,297,412	pyoverdin	2%

## Data Availability

Not applicable.

## Supplementary Materials

The following are available online at https://www.mdpi.com/article/10.3390/pathogens10111423/s1, Figure S1: hydrolytic enzymes activities, Figure S2: plant growth promoting attributes; Table S1: Statistics of open reading frame (OFR) predictions, Table S2: Statistics of non-coding RNA predictions, Table S3: CRISPRs prediction results.
