# Supplementary material for: In Vitro Study of Biocontrol Potential of Rhizospheric Pseudomonas aeruginosa against Pathogenic Fungi of Saffron (Crocus sativus L.)
Source: Pathogens. 2021 Nov 2;10(11):1423. doi: 10.3390/pathogens10111423 (PMC8620626; doi:10.3390/pathogens10111423)
Supplement: Supplementary file 1 [file pathogens-10-01423-s001.zip › pathogens-1355965-supplementary.pdf]

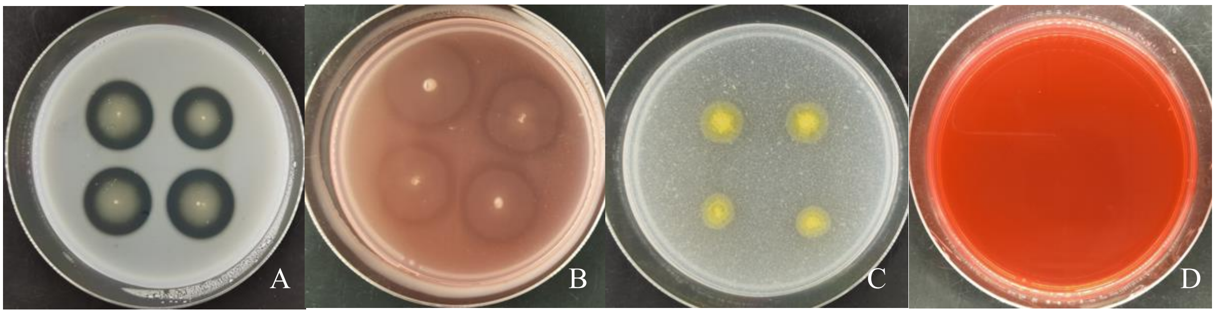

Figure S1. hydrolytic enzymes activities

(A: protease +, B: glucanase +, C: chitinase –, D: cellulose –)

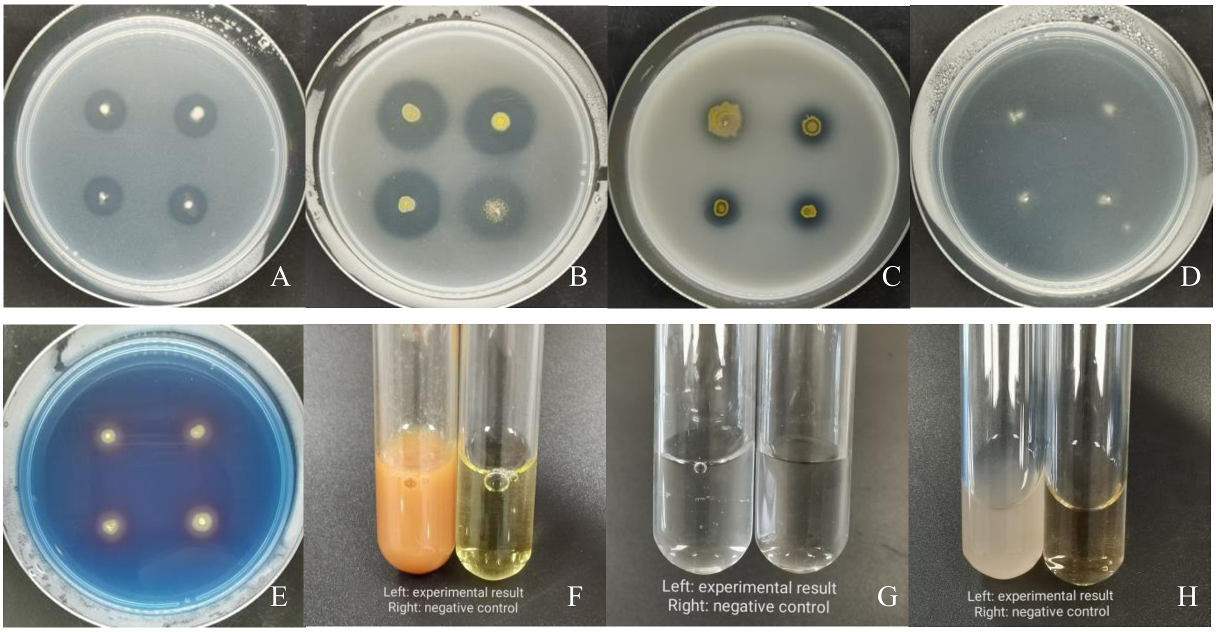

Figure S2. plant growth promoting attributes

(A: nitrogen fixation +, B: organic phosphate solubilization +, C: inorganic phosphate solubilization +, D: potassium dissolvion –, E: siderophore production +, F: NH_3 production +, G: ACC deaminase enzyme –, H: IAA production –)

Table S1 Statistics of open reading frame (OFR) predictions

Property	Value
ORF num	5809
ORF total length	5641239 bp
ORF density	0.910 genes per kb
Longest ORF length	14976 bp
ORF average length	971.12 bp
Intergenetic region length	741106 bp
ORF/Genome(coding percentage)	88.39%
Intergenetic length/Genome	11.61%
GC content in ORF region	67.24%
GC content in intergenetic region	60.60%

Table S2 Statistics of non-coding RNA predictions

Type	Copy Number	Avg. length (bp)	Total length (bp)	percent of genome (%)
5S rRNA	4	110	440	0.0069
16S rRNA	4	1531	6,124	0.0960
23S rRNA	4	2888	11,552	0.1810
tRNA	64	78	5,018	0.0786
ncRNA	75	50	11,316	0.1773

Table S3 CRISPRs prediction results

CRISPRs type	Start	End	Number of Spacer	Length (bp)	Genome %
Confirmed	1,509,057	1,509,624	9	567	0.0089
Confirmed	2,870,988	2,872,271	21	1,283	0.0201
Confirmed	2,881,146	2,882,434	21	1,288	0.0202
Confirmed	331,258	331,371	1	113	0.0018
Confirmed	1,667,569	1,667,719	1	150	0.0024
Confirmed	2,420,690	2,420,783	1	93	0.0015
Confirmed	5,906,018	5,906,118	1	100	0.0016
